# The vestibulo-ocular and vestibulospinal reflexes minimally impact the freezing of gait in patients with early-to-moderate Parkinson’s disease

**DOI:** 10.1016/j.prdoa.2025.100319

**Published:** 2025-04-06

**Authors:** Yukang Kim, Tonghoon Woo, Hanseob Kim, Kyoungwon Baik, Sun-Uk Lee, Chan-Nyoung Lee, Gerard J. Kim, Seoui Kwag, Hyunsoh Park, Ji-Soo Kim, Kun-Woo Park

**Affiliations:** a Neurotology and Neuro-ophthalmology Laboratory, Korea University Medical Center, Seoul, South Korea; b Department of Computer Science and Engineering, Korea University, Seoul, South Korea; c Department of Neurology, Korea University Medical Center, Seoul, South Korea; d Department of Neurology, Seoul National University College of Medicine, Seoul, South Korea; e Dizziness Center, Clinical Neuroscience Center, Seoul National University Bundang Hospital, Seongnam, South Korea

**Keywords:** Parkinson’s disease, Vertigo, Head-impulse test, Nystagmus

## Abstract

**Introduction:**

Freezing of gait (FOG) is a disabling condition that often leads to falls and severe postural instability in patients with Parkinson’s disease (PD). This study aimed to determine whether FOG is associated with the integrity of the vestibulo-ocular (VOR) and vestibulospinal reflexes (VSR).

**Methods:**

We retrospectively collected 138 patients with de novo PD at a tertiary medical center between February 2022 and February 2025. Each patient was queried and assessed for FOG status during the initial assessment. All patients underwent video head-impulse tests (video-HIT), cervical vestibular-evoked myogenic potential (cVEMP), ocular vestibular-evoked myogenic potential (oVEMP), and motion analysis.

**Results:**

FOG was observed in 23 patients (23/138, 17 %). The head impulse gain of the VOR did not differ between freezers and non-freezers in any semicircular canal. The new FOG questionnaire score showed no correlation with the VOR gain for any canal. The oVEMP and cVEMP parameters did not differ between freezers and non-freezers either. Multivariable logistic regression analysis revealed a positive association between FOG and MDS-UPDRS-III (*p =* 0.016). However, FOG was not associated with the VOR gain in any canal or abnormalities on oVEMP or cVEMP.

**Conclusions:**

Our preliminary data suggest that FOG is associated with the severity of motor symptoms in patients with early-to-moderate PD. While the integrity of the VOR or VSR is not currently associated with FOG, a well-designed future study could provide more nuanced insights into the relationship with these factors.

## Highlights

• Although former studies have suggested a possible link between vestibular deficits and freezing of gait (FOG), their relationship requires further elucidation.
• FOG is associated with the severity of motor symptoms in patients with Parkinson’s disease (PD), regardless of the results of video head-impulse tests and vestibular-evoked myogenic potentials.
• While the integrity of the VOR or VSR is not currently associated with FOG, a well-designed future study could provide more nuanced insights into the relationship with these factors.

## 1 Introduction

Freezing of gait (FOG) refers to an episodic inability to initiate locomotion, characterized by disturbed stepping patterns that respond to focused attention and visual cues [1]. FOG is observed in approximately 27 % of patients during the early stage of Parkinson’s disease (PD), increasing to up to 86 % in the advanced stages [2]. Due to its sudden and unpredictable nature, FOG often leads to falls and injuries [1]. Unlike cardinal motor symptoms, FOG tends to respond poorly to dopaminergic medication [1].

The association between vestibular dysfunction and the FOG remains unclear in PD. Earlier studies using posturography have suggested a possible connection [3]: Freezers fail to maintain balance during Romberg condition 4, which relies primarily on vestibular input, despite passing conditions 1–3 in PD. However, objective vestibular testing has been not applied to confirm failure during Romberg condition 4 in prior studies.

Recently, we established a prospective patient cohort and conducted a thorough vestibular evaluation in consecutive patients with de-novo PD. We found that video head-impulse tests (video-HITs), cervical vestibular evoked myogenic potentials (cVEMP), and ocular vestibular evoked myogenic potentials (oVEMP) were impaired in approximately one-third of the patients with early-to-moderate PD. However, their clinical significance may be minimal for maintaining balance, as falls are primarily influenced by the overall clinical severity of PD [4,5]. Similar to falling, FOG also limits patients' daily activity. This similarity provides a chance to investigate how FOG is related to vestibular function. Clarifying the relationship with FOG is crucial because it could help assess whether novel vestibular rehabilitation, caloric, or galvanic stimulation might be effective for managing FOG in PD. This study aimed to determine whether FOG is associated with the integrity of the vestibulo-ocular (VOR) and vestibulospinal reflexes (VSR).

## 2 Methods

### 2.1 Patients and vestibular evaluation

This nested case-control study was originally collected to investigate the association between falling, VOR, and VSR among patients recruited between February 2022 and May 2024 [4,5]. We further collected data from May 2024 to February 2025 to investigate the association of FOG, VOR, and VSR. Finally, we retrospectively analyzed the data of 138 patients (mean age ± standard deviation [SD] = 70 ± 10 years; 67 females).

Head and eye movements were recorded using video-HITs (SLVNG, SLMED, Seoul, South Korea), as described previously [5]. The results of video-HITs were considered positive (abnormal) if 1) they fell outside the normal VOR gain range established from 30 healthy age-, sex-matched participants (mean age ± SD = 70 ± 9 years, 15 males; normal gain for the horizontal canal [HC] = 0.81–1.33; normal gain for the anterior canal [AC] = 0.70 – 1.32; normal gain for the posterior canal [PC] = 0.61–1.34) and 2) greater cumulative saccadic amplitude (> 3.2 ${\;}^{{}^{\circ}}$ for the HC; > 0.6 ${\;}^{{}^{\circ}}$ for the AC; and > 2.9 ${\;}^{{}^{\circ}}$ for the PC). Corrective saccades were defined as being present when they occurred with the peak eye velocity exceeding 60 ${\;}^{{}^{\circ}}$/s with the cut-off value decided by the main sequence of saccades, as described previously [6].

cVEMP and oVEMP were recorded as described previously by the same examiner using a Nicolet Viking Select unit (Nicolet-Biomedical, Madison, WI, USA).

oVEMPs were elicited by tapping the hairline at the AFz using an electric reflex hammer (VIASYS Healthcare, CA, USA). Bilateral responses were recorded simultaneously after applying the tapping stimuli. Up to 60 tapping stimuli were applied at a 2 Hz frequency and approximately 0.45 g of force. The responses were averaged for each test, and the average latency of the initial negative peak (n1) and n1–p1 amplitude were determined. oVEMP responses were obtained at least twice, and the mean was calculated. The interaural difference (IAD, %) of the oVEMP amplitudes was calculated as [100 × (A_Right_ − A_Left_)/(A_right_ + A_left_)].

cVEMPs were recorded with the patient lying supine on a bed, with the head raised by approximately 30° and rotated to one side to contract the sternocleidomastoid muscle (SCM). A short burst of alternating tone (110 dB nHL, 123.5 dB SPL, 500 Hz, rise time = 2 ms, plateau = 3 ms, and fall time = 2 ms) was applied at a 2.1 Hz frequency monoaurally via headphones. The signal was sampled (48 kHz), amplified, and bandpass-filtered at 30–1500 Hz. No rectification or smoothing was performed while the cVEMP responses were recorded. cVEMP responses for up to 80 stimuli were averaged for each test. Responses were obtained at least twice for each ear, and the mean values were calculated.

Absolute cVEMP amplitudes were normalized and divided by the mean tonic activation of the SCM during recording. To compare the normalized p13–n23 amplitudes of cVEMP between the right and left sides, the IAD (%) was calculated. The peak latency of p13 was also calculated. To establish the reference ranges, oVEMP and cVEMP responses were assessed in 30 healthy participants (mean age ± SD = 69 ± 10 years, 15 males) with no prior history of auditory or vestibular disorders (reference range for oVEMP; n1 latency < 8.74 ms, IAD < 24.2 %; reference range for cVEMP; p13 latency < 19.8 ms, normalized p13–n23 amplitude > 0.97 μV, IAD < 31.7 %). Unilateral abnormalities were defined as a delayed and/or decreased (or absent) response in one ear, while bilateral abnormalities were defined as a delayed and/or decreased (or absent) response in both ears. Patients showing absent responses during cVEMP were further evaluated via forehead tapping, tympanoscopic examination, pure tone, and speech audiometry.

### 2.2 Assessment of FOG

FOG was defined as present if 1) patients reported experiencing FOG at least once during the past month based on item 2.13 of the MDS-UPDRS (≥ 1), 2) one or more FOG episodes were provoked and observed by two experienced movement specialists (K.B. and C.N.L, independently, but not multiple rating) during the experiment, or 3) FOG was detected during motion analysis. To quantify the severity of FOG, the new FOG questionnaire was administered to patients identified as having FOG [7].

### 2.3 Motion analysis

Temporal and spatial gait characteristics were measured using a gait analyzer (GaitRite. Twelve fit AP1105; CIR Systems, Franklin, NJ, USA) [4,5]. The patients were instructed to walk along an 8.3-meter-long and 0.89-meter-wide walkway. Patients turned their bodies after completing one cycle, which was not included in the analysis to isolate straight-path gait characteristics. Six gait cycles were measured, and the average values were calculated, excluding the initial and final cycles. The walking pattern from heel stroke to toe-off was collected using pressure sensors embedded into the carpet, and the temporal and spatial gait parameters were recorded in each patient. FOG episodes were defined as unintentional and temporary instances where the feet failed to move for more than 1 s while initiating movement or walking. The total number of freezing episodes was recorded during motion analysis.

### 2.4 Statistical analysis

Nominal and independent variables were compared using the χ ^2^ or Fisher’s exact test. Continuous and independent variables were compared using the Student’s *t*-test, Mann–Whitney *U* test, and Spearman’s correlation. For logistic regression analysis, significant variables were selected using the backward variable selection method. Statistical significance was set at *p* < 0.05 in the multivariable logistic regression analyses.

Statistical analyses were performed using the R software package (version 3.4.0; https://www.r-project.org), and statistical significance was set at two-tailed *p* < 0.05. Mediation analysis, including the interaction effects between variables, was performed using SAS version 9.4 (SAS Institute Inc., Cary, NC).

## 3 Results

### 3.1 Clinical characteristics

The detailed clinical characteristics of the 138 patients are presented in the S upplementary Table S1. Findings from 79 patients have been previously reported [4,5].

FOG was observed in 23 patients (23/138, 17 %). Freezing was documented during motion analysis in seven (9/23, 39 %), of which the freezing episodes lasted from 3.4 to 11.5 s (median = 4.6 s, IQR = 4.0–6.2). The new-FOG questionnaire score ranged from 0 to 25 (median = 3, IQR = 0–9). Notably, eight out of 23 patients (35 %) scored 0 despite FOG being detected during examination or motion analysis.

### 3.2 Video-HITs

The VOR gain was normal in 78 patients with PD (78/138, 57 %; Table 1 and S upplementary Table). However, in 29 patients (21 %), there was at least one one VOR gain decrease as follows: any HC in 17 (left only, n = 3; right only, n = 6; both left and right, n = 8), any AC in 16 (left only, n = 3; right only, n = 9; both left and right, n = 4), or any PC in 10 (left only, n = 4; right only, n = 2, both left and right abnormal, n = 4). Fifteen patients showed a VOR gain decrease in isolated canals, and fourteen showed a decrease in multiple canals. Thirty-seven patients (27 %) exhibited an overestimation of the VOR gain in at least one canal plane.

**Table 1:** Clinical characteristics and results of the evaluation between the patients with and without freezing.

	**Freezers (n = 23)**	**Non-freezers (n = 115)**	***p* value**
Age, mean ± SD, years	75 ± 11	69 ± 10	*0.004*
Sex, female (%)	15 (65)	52 (45)	0.080
Body weight, mean ± SD, kg	59 ± 9	64 ± 10	*0.043*
Disease duration, median (IQR), months^a^	24 (12–36)	12 (7–27)	*0.042*
MDS-UPDRS-III, median (IQR)	31 (26–50)	25 (17–33)	*0.003*
H&Y scale, median (IQR)	2.5 (2–3)	2 (2–2.5)	*0.008*
MMSE	27 (24–29)	27 (25–29)	0.418
RBD	8 (35)	48 (42)	0.535
Orthostatic hypotension	5 (39)	58 (51)	0.304
New-FOG questionnaire score	0 (0–7)	−	
Decreased VOR gain	10 (44)	49 (43)	0.939
**Video-HITs, median (IQR)**			
*VOR gain, HC*	1.06 (0.92–1.11)	1.05 (0.97–1.16)	0.560
*VOR gain, AC*	0.97 (0.89–1.18)	1.07 (0.93–1.20)	0.399
*VOR gain, PC*	1.00 (0.85–1.11)	1.01 (0.91–1.16)	0.419
**oVEMP abnormality**			0.483
*Normal*	14 (74)	84 (80)	
*Unilateral*	4 (21)	12 (11)	
*Bilateral*	1 (5)	9 (9)	
oVEMP parameters			
*n1 latency*	7.3 (6.7–7.9)	7.3 (6.7–7.7)	0.652
*n1–p1 amplitude*	7.3 (5.5–11.1)	7.0 (4.5–10.0)	0.589
*IAD, %*^b^	4.8 (0.6–20.6)	4.0 (0–11.9)	0.654
**cVEMP abnormality**^c^			0.483
*Normal*	14 (74)	84 (80)	
*Unilateral*	4 (21)	12 (11)	
*Bilateral*	1 (5)	9 (9)	
cVEMP parameters			
*p13 latency*	16.0 (14.6–17.6)	15.7 (15.1–16.9)	0.832
*Normalized p13–n23 amplitude*	1.36 (1.21–1.89)	1.89 (1.34–2.52)	0.056
*IAD, %*^b^	9.1 (4.2–14.9)	8.7 (4.1–15.3)	0.778
**Motion analyses, mean**±**SD**			
*Walking velocity, cm/s*	69.2 (52.2–93.5)	85.4 (70.9–104.2)	*0.009*
*Walking cadence, step/*min	104.6 (97.1–117.5)	106.1 (97.2–115.5)	0.662
*Step length difference, cm*	2.8 (1.6–3.6)	2.0 (0.7–3.5)	0.603
*Double support time, %gait cycle*	30.9 (25.8–35.0)	26.5 (23.9–30.6)	*0.043*
**Comorbidities**			
*Diabetes mellitus (%)*	7 (30)	28 (24)	0.540
*Hypertension (%)*	15 (65)	55 (48)	0.128
*Dyslipidemia (%)*	11 (48)	45 (39)	0.438
*Cerebrovascular attack (%)*	2 (9)	12 (10)	>0.999
*Coronary artery occlusive disease (%)*	2 (9)	14 (12)	>0.999
*Depression (%)*	5 (22)	19 (17)	0.547
*Anxiety (%)*	3 (13)	10 (9)	0.455

AC = anterior canal, cVEMP = cervical vestibular-evoked myogenic potential, FOG = freezing of gait, HC = horizontal canal, H&Y = Hoehn and Yahr, IAD = interaural difference, IQR = interquartile range, MDS-UPDRS-III = Movement Disorder Society-Unified Parkinson’s Disease Rating Scale motor part, MMSE = mini-mental state examination, oVEMP = ocular VEMP, PC = posterior canal, REM = Rapid eye movement, SD = standard deviation, VOR = vestibulo-ocular reflex.

a Disease duration refers to the period from onset of motor symptoms to presentation

b Absolute values.

c Excluding 14 patients with poor steinocleidomastoid contraction

The VOR gain did not differ for any canal between the freezers and non-freezers (*p* = 0.560 for HC; *p* = 0.399 for AC; *p* = 0.419 for PC; Table 1). The new FOG questionnaire score showed no correlation with VOR gain for any canal (*r* = -0.331, *p* = 0.123 for HC; *r* = 0.059., *p* = 0.790 for AC; *r* = 0.074, *p* = 0.738 for PC). Among patients with VOR decrease (n = 29), the new FOG questionnaire did not correlate with VOR gain for any individual canal (*r* = -0.500, *p* = 0.391 for the right HC; *r* = -0.600, *p* = 0.285 for the left HC; *r* = -0.400, *p* = 0.505 for the right AC; *r* = -0.600, *p* = 0.285 for the left AC; *r* = -0.600, *p* = 0.285 for the right PC; *r* = -0.103, *p* = 0.870 for the left PC).

### 3.3 cVEMP and oVEMP

cVEMP was normal in 98 out of 124 patients with PD (79 %, excluding 14 patients with inadequate sternocleidomastoid contraction); however, 16 patients showed unilateral abnormalities, and 10 exhibited bilateral abnormalities. Responses were decreased in six, absent in 20, and delayed in 10 ears being stimulated. The oVEMP was normal in 91 out of 138 patients with PD (66 %); however, 23 patients showed unilateral abnormalities, and 24 exhibited bilateral abnormalities. Responses were decreased in 16, absent in 47, delayed in 5, and both decreased and delayed in 3 ears being stimulated.

None of the oVEMP or cVEMP parameters differed between the two groups. (Table 1). Among patients showing FOG, the new FOG questionnaire score negatively correlated with p13 latency (*r* = -0.529, *p* = 0.035). Otherwise, the new FOG questionnaire score showed no correlation with cVEMP (*r* = -0.278, *p* = 0.249 for normalized p13–n23 amplitude; *r* = 0.322, *p* = 0.179 for IAD) or oVEMP parameters (*r* = -0.231, *p* = 0.327 for n1 latency; *r* = -0.312, *p* = 0.148 for n1–p1 amplitude; *r* = -0.079, *p* = 0.728 for IAD).

### 3.4 Motion analysis

Freezers demonstrated a slower walking velocity (*p* = 0.009) and a longer double support time than non-freezers (*p* = 0.043; Table 1).

The new FOG questionnaire score positively correlated with double support time (*r* = 0.523, *p* = 0.018). However, no correlation was found with cadence (*r* = -0.109, *p* = 0.285), velocity (*r* = -0.394, *p* = 0.085), step length difference (*r* = 0.350, *p* = 0.131), or freezing time during motion analysis (*r* = 0.313, *p* = 0.179).

### 3.5 Prediction of FOG

Multivariable logistic regression analysis revealed a positive association between FOG and MDS-UPDRS-III (*p =* 0.016; Table 2). However, FOG was not associated with the VOR gain in any canal, oVEMP abnormalities, or cVEMP abnormalities.

**Table 2:** Prediction of FOG using multiple logistic regression analyses.

Variables	Univariate analysis		Multivariable analysis^b^	
	**OR (95 % CI)**	***p*-value**	**OR (95 % CI)**	***p*-value**
Age, years	1.08 (1.02–1.15)	*0.007*		
Female sex	2.27 (0.89–5.78)	0.085		
Body weight, kg	0.95 (0.91–1.00)	*0.046*	0.94 (0.88–1.00)	0.060
Disease duration, months	1.01 (0.99–1.02)	0.425		
MMSE	0.95 (0.84–1.07)	0.410	1.21 (0.98–1.49)	0.082
H&Y ≥ 3.0	3.46 (1.30–9.20)	*0.013*		
MDS-UPDRS-III	1.06 (1.02–1.09)	*0.001*	1.05 (1.01–1.10)	*0.016*
VOR gain, HC^c^	0.18 (0.02–1.98)	0.160		
VOR gain, AC^c^	0.30 (0.04–2.26)	0.242		
VOR gain, PC^c^	0.33 (0.05–2.35)	0.267		
oVEMP abnormality		0.409		
*Unilateral*	2.12 (0.71–6.37)	0.181		
*Bilateral*	1.20 (0.35–4.08)	0.770		
cVEMP abnormality		0.495		
*Unilateral*	2.00 (0.56–7.09)	0.283		
*Bilateral*	0.67 (0.08–5.68)	0.711		
Double support time, second	1.09 (1.01–1.19)	*0.027*	1.10 (0.99–1.23)	0.090

AC = anterior canal, CI = confidence interval, cVEMP = cervical vestibular-evoked myogenic potential, FOG = freezing of gait, HC = horizontal canal, H&Y = Hoehn and Yahr, IAD = interaural difference, IQR = interquartile range, MDS-UPDRS-III = Movement Disorder Society-Unified Parkinson’s Disease Rating Scale motor part, MMSE = mini-mental state examination, oVEMP = ocular VEMP, PC = posterior canal, REM = Rapid eye movement, SD = standard deviation, VOR = vestibulo-ocular reflex.

^a^Disease duration refers to the period from onset of motor symptoms to presentation.

b For logistic regression analysis, significant variables were selected using the backward variable selection method.

c Due to the potential bias introduced by different cut-off points for VOR gain across the three canals, VOR gain was chosen as the continuous input variable rather than a trichotomized variable of normal, decreased, or increased.

## 4 Discussion

The findings of this study can be summarized as follows: 1) FOG was associated with MDS-UPDRS-III; 2) in contrast, no association was found between FOG and VOR gain in any canal, oVEMP abnormalities, or cVEMP abnormalities; 3) no correlation was found between the new-FOG questionnaire score and VOR gain in any canal, oVEMP, or cVEMP parameters.

### 4.1 Theoretical background for the connection between the vestibular system and FOG

Failure to integrate postural sensory inputs may contribute to the development of FOG [8]. Given that the FOG can be triggered in situations where sensory inputs conflict and balance is challenged: FOG becomes apparent when simulating difficulty processing vestibular sensory information during postural perturbation or providing visual misinformation [3,9].

The pedunculopontine nucleus (PPN) and mesencephalic reticular formation (MRF) —which have an established role in gait initiation— send projections and receive input from neural substrates to process the central vestibular pathway, suggesting a potential link between vestibular function and FOG. Single-cell recordings demonstrate that most PPN cells respond to rotation and translation stimuli [10]. A projection from the vestibular nuclei to the PPN has been reported in rodents [11]. The neural connection between the fastigial nucleus, PPN, and central MRF would likely be the primary source of vestibular input [11]. The interaction between vestibular dysfunction and FOG has been investigated in this context. Notably, subtle PPN dysfunction explains why only a small subset of PD patients exhibit abnormal vestibular reflexes in our study. However, clinical evidence has been slow to emerge.

### 4.2 Clinical evidence relating FOG with vestibular function

Previously, Huh YE et al. found that patients with PD and FOG exhibited worse performance on posturography than those without FOG, especially during perturbed somatosensory input (i.e., Romberg condition 4–6) [3]. Similarly, patients with FOG tend to fail during Romberg condition 4, which simulates a balance challenge relying primarily on vestibular inputs [12]. These results suggest that FOG arises from compromised postural sensory processing, particularly under distorted somatosensory conditions [12]. However, FOG was observed only in patients with a disease duration of 5–10 years, where the severity of motor symptoms was strictly controlled, preventing assessment of its impact on vestibular abnormalities [3,12]. Most importantly, previous studies often omitted objective vestibular testings, e.g., video-HITs, cVEMP, and oVEMP, leaving a gap in understanding failure during Romberg condition 4 in PD freezers. Our study implicates that scrutinized neurotologic investigation could allow futher discussion on one of major symptoms of the disorder.

Recently, Jiang Y et al. reported oVEMP responses are more frequently absent in freezers than in non-freezers (78 % vs. 31 %) [13], likely due to the direct involvement of the central vestibular pathway by synucleinopathy. However, the brain areas controlling eye movements are generally less affected by alpha-synuclein deposits (Lewy bodies) compared to other brainstem nuclei, at least until the later stages of the disease [14]. In fact, there are conflicting studies on whether cVEMP and oVEMP are affected by PD, showing large variablity across studies [15]. This heterogeneity may result from different stimulation methods and patient populations across studies. Most of all, given that sound stimulation often fails to invoke VEMP responses in old age, another stimulation method should be applied for direct comparison. The n1 response obtained through forehead tapping in our study exhibited a relatively shorter latency than other stimulation methods. Thus, future studies are warranted to validate any potential association between FOG and VEMP parameters to generalize our findings.

### 4.3 The VOR and VSR for balance in PD: Implications for FOG

Our findings suggest that afferent information processing does not determine FOG in patients with mild-to-moderate PD. Our study shows conflicting results with prior studies that shows vestibular contribution on FOG [12]. On the Romberg condition 4, wherein one has to maintain standing relying mostly on vestibular information, PD freezers are significantly more likely to fall than nonfreezers, suggesting a more pronounced vestibular balance control deficit among freezers relative to non-freezers [12]. This vestibular contribution on FOG may mediated from causing postural instability, given a greater bodily sway during Romberg condition 4 among those with postural instability. This association implies that vestibular balance control deficit might be a strong contributing factor to postural instability in PD [16]. However, our mediation analysis did not show that vestibular dysfunction contributes indirectly to FOG via interactions with postural instability as well as being a direct causative factor.

Our result should be interpreted with caution. The negative association between the electrophysiologic tests and FOG may not necessarily imply a lack of vestibular contribution to FOG. The ascending vestibular pathway is usually preserved in early-to-moderate PD. The diagnostic yield of video-HITs, oVEMP, and cVEMP regarding multisensory integration is relatively limited [17]. Furthermore, sensory re-weighting can be poor despite intact vestibular inputs in PD [9,18]. Video-HITs, cVEMP, and oVEMP are specialized in assessing the integrity of VOR and VSR, but these tests cannot estimate the interaction with different sensory modalities. In this context, direct vestibular modulation (e.g., galvanic or caloric vestibular stimulation) or functional imaging that enables estimation of the interaction with other sensory or motor subdomain [19].

### 4.4 Measure of FOG

In addition to clinical examinations and FOG questionnaires, motion analysis was performed as an objective measure to complement the clinical diagnosis of FOG in our study. However, FOG usually occurs during step initiation while turning or changing directions, and motion analysis is not optimal for evaluating FOG. The currently adopted gold standard for quantifying FOG is through video scoring of timed up and go trials by independent and multiple experts [20]. The recent adoption of an automatic video scoring algorithm can be promising for quantifying FOG [21]. Likewise, kinematic analysis using a motion-capture system or wearable sensors also allows for the detection of FOG episodes [1,2]. The discrepancies between the new-FOG questionnaire and motion analysis highlight the need for an objective FOG measurement in our patients. However, negative associations between FOG and neurotologic tests have been consistently observed across different modalities. Our findings also underscore the need for future studies to explore these effects in the advanced stages of PD, in which FOG is far more prevalent.

### 4.5 Caveats and limitations of our study and suggestions for future studies

Our investigation was based on a registry of patients with mild-to-moderate de novo PD, with FOG observed in only 17 % of our patient cohort. Therefore, we could not determine whether the findings may differ in those with advanced PD. Another major limitation was that the FOG was assessed primarily through a questionnaire, which is subject to selection and recall biases. As aforementioned, although motion analysis complemented the detection of the FOG, they are also not ideal for accurate measurements. We adopted a study protocol for walking paths to analyze straight-path gait characteristics and excluded turning. Since FOG often occurs during turning and step initiation, wearable sensors or inertial measurement units can provide more detailed temporal data on FOG episodes [1,2]. Additionally, video-HIT, cVEMP, and oVEMP may not be sensitive enough to detect subtle vestibular impairment or estimate the interaction with other sensory or motor domains causing FOG. The fact that FOG tends not to respond to levodopa suggests that other afferent pathway sensory integration may be involved in developing FOG. In this context, galvanic stimulation or functional MRI during vestibular stimulation can aid in further understanding the contribution of VOR and VSR on FOG. A translational approach of applying established FOG treatment modalities such as sensory cueing, gait training, and feedback to patients with different VOR and VSR functions may provide valuable insight [22]. Thus, our findings are preliminary and require future validation with ideal design and diagnostic modalities.

In conclusion, our preliminary data suggest that FOG is associated with the severity of motor symptoms in patients with early-to-moderate PD. While the integrity of the VOR or VSR is not currently associated with FOG, a well-designed future study could provide more nuanced insights into the relationship with these factors.

## CRediT authorship contribution statement

**Yukang Kim:** Writing – original draft, Investigation, Formal analysis. **Tonghoon Woo:** Writing – original draft, Formal analysis. **Hanseob Kim:** Writing – review & editing, Methodology. **Kyoungwon Baik:** Investigation, Formal analysis, Conceptualization. **Sun-Uk Lee:** Writing – review & editing, Writing – original draft, Visualization, Supervision, Methodology, Funding acquisition, Formal analysis, Data curation, Conceptualization. **Chan-Nyoung Lee:** Supervision, Investigation. **Gerard J. Kim:** Writing – review & editing. **Seoui Kwag:** Investigation. **Hyunsoh Park:** Investigation. **Ji-Soo Kim:** Writing – review & editing, Supervision. **Kun-Woo Park:** Investigation, Formal analysis, Data curation.

## Declaration of competing interest

The authors declare that they have no known competing financial interests or personal relationships that could have appeared to influence the work reported in this paper.

Drs. Y. Kim, D. Woo, H. Kim, S.U. Lee, K. Baik, C.N. Lee, G. Kim, S. Kwag, H. Park, and K.W. Park report no disclosures.
