# Supplementary material for: The vestibulo-ocular and vestibulospinal reflexes minimally impact the freezing of gait in patients with early-to-moderate Parkinson’s disease
Source: Clin Park Relat Disord. 2025 Apr 6;12:100319. doi: 10.1016/j.prdoa.2025.100319 (PMC12008544; doi:10.1016/j.prdoa.2025.100319)
Supplement: Supplementary Data 1 [file mmc1.docx]

**Supplementary table 1.** Clinical characteristics of the patients

| **Variables** | **Values** |
| --- | --- |
| Age, mean$\pm$SD, years | 70$\pm$10 |
| Sex, female (%) | 67 (49) |
| Body weight, mean$\pm$SD, kg | 63$\pm$10 |
| Disease duration, median (IQR), months^a^ | 12 (7–30) |
| MDS-UPDRS-III, median (IQR) | 26 (18–35) |
| MMSE | 27 (25–29) |
| H&Y scale (%) |  |
| 1.0 | 29 (21) |
| 1.5 | 2 (1) |
| 2 | 51 (37) |
| 2.5 | 29 (21) |
| 3 | 27 (19.5) |
| VOR gain, mean$\pm$SD |  |
| Right HC | 1.09$\pm$0.23 |
| Left HC | 1.04$\pm$0.21 |
| Right AC | 1.05$\pm$0.29 |
| Left AC | 1.08$\pm$0.25 |
| Right PC | 1.04$\pm$0.27 |
| Left PC | 1.00$\pm$0.26 |
| oVEMP |  |
| n1 latency, ms | 7.3 (6.7–7.7) |
| n1–p1 amplitude, $\mu$V | 7.0 (4.6–10.5) |
| IAD, %^b^ | 4.0 (0–10.2) |
| cVEMP |  |
| p13 latency, ms | 15.8 (15.1–17.2) |
| Normalized p13–n23 amplitude, $\mu$V | 1.84 (1.26–2.34) |
| IAD, %^b^ | 8.7 (3.7–15.3) |
| Orthostatic hypotension (%) | 67 (49) |
| RBD (%) | 56 (41) |
| FOG (%) | 23 (17) |

^a^Disease duration refers to the period from onset of motor symptoms to presentation

^b^Absolute values

AC = anterior canal, cVEMP = cervical vestibular-evoked myogenic potential, FOG = freezing of gait, HC = horizontal canal, H&Y = Hoehn and Yahr, IAD = interaural difference, IQR = interquartile range, MDS-UPDRS-III = Movement Disorder Society-Unified Parkinson’s Disease Rating Scale motor part, MMSE = mini-mental state examination, oVEMP = ocular VEMP, PC = posterior canal, RBD = Rapid eye movement sleep behavior disorder, SD = standard deviation, VOR = vestibulo-ocular reflex

**Supplementary table 2. Mediation analysis of the variables for causing FOG through postural instability***

| Variable (X) |  | **Estimate** | **Standard**  **Error** | **Wald 95%**  **Confidence Limits** | | **Z** | **Pr > \|Z\|** |
| --- | --- | --- | --- | --- | --- | --- | --- |
| VOR gain, HC | **Odds Ratio Total Effect** | 0.1827 | 0.2198 | -0.248 | 0.6135 | -3.72 | 0.0002 |
|  | **Odds Ratio Controlled Direct Effect (CDE)** | 0.3928 | 0.6359 | -0.8536 | 1.6391 | -0.95 | 0.3396 |
|  | **Odds Ratio Natural Direct Effect (NDE)** | 0.2856 | 0.3997 | -0.4978 | 1.0689 | -1.79 | 0.0739 |
|  | **Odds Ratio Natural Indirect Effect (NIE)** | 0.6398 | 0.5509 | -0.44 | 1.7197 | -0.65 | 0.5133 |
| VOR gain, AC | **Odds Ratio Total Effect** | 0.1915 | 0.2383 | -0.2757 | 0.6586 | -3.39 | 0.0007 |
|  | **Odds Ratio Controlled Direct Effect (CDE)** | 0.8766 | 1.0994 | -1.2781 | 3.0314 | -0.11 | 0.9107 |
|  | **Odds Ratio Natural Direct Effect (NDE)** | 0.1983 | 0.2471 | -0.2861 | 0.6826 | -3.24 | 0.0012 |
|  | **Odds Ratio Natural Indirect Effect (NIE)** | 0.9657 | 0.09126 | 0.7868 | 1.1446 | -0.38 | 0.7071 |
| VOR gain, PC | **Odds Ratio Total Effect** | 0.241 | 0.2788 | -0.3054 | 0.7874 | -2.72 | 0.0065 |
|  | **Odds Ratio Controlled Direct Effect (CDE)** | 0.6845 | 0.8417 | -0.9653 | 2.3342 | -0.37 | 0.7078 |
|  | **Odds Ratio Natural Direct Effect (NDE)** | 0.2408 | 0.28 | -0.3079 | 0.7896 | -2.71 | 0.0067 |
|  | **Odds Ratio Natural Indirect Effect (NIE)** | 1.0006 | 0.01757 | 0.9662 | 1.0351 | 0.04 | 0.9709 |
| oVEMP abnormality | **Odds Ratio Total Effect** | 1.0216 | 0.3236 | 0.3874 | 1.6558 | 0.07 | 0.9467 |
|  | **Odds Ratio Controlled Direct Effect (CDE)** | 1.4644 | 0.4916 | 0.5009 | 2.4278 | 0.94 | 0.3448 |
|  | **Odds Ratio Natural Direct Effect (NDE)** | 0.9947 | 0.3086 | 0.3898 | 1.5995 | -0.02 | 0.9862 |
|  | **Odds Ratio Natural Indirect Effect (NIE)** | 1.0271 | 0.04933 | 0.9304 | 1.1238 | 0.55 | 0.5827 |
| cVEMP abnormality | **Odds Ratio Total Effect** | 1.2512 | 0.5438 | 0.1853 | 2.3171 | 0.46 | 0.6442 |
|  | **Odds Ratio Controlled Direct Effect (CDE)** | 1.969 | 0.7614 | 0.4767 | 3.4614 | 1.27 | 0.2031 |
|  | **Odds Ratio Natural Direct Effect (NDE)** | 1.2066 | 0.5067 | 0.2136 | 2.1997 | 0.41 | 0.6834 |
|  | **Odds Ratio Natural Indirect Effect (NIE)** | 1.0369 | 0.07188 | 0.896 | 1.1778 | 0.51 | 0.6074 |
| n1 latency | **Odds Ratio Total Effect** | 0.591 | 0.3854 | -0.1644 | 1.3463 | -1.06 | 0.2885 |
|  | **Odds Ratio Controlled Direct Effect (CDE)** | 1.1436 | 0.3468 | 0.4638 | 1.8233 | 0.41 | 0.6789 |
|  | **Odds Ratio Natural Direct Effect (NDE)** | 0.5452 | 0.3475 | -0.1358 | 1.2262 | -1.31 | 0.1906 |
|  | **Odds Ratio Natural Indirect Effect (NIE)** | 1.0839 | 0.08931 | 0.9088 | 1.2589 | 0.94 | 0.3476 |
| n1–p1 amplitude | **Odds Ratio Total Effect** | 0.9952 | 0.04387 | 0.9092 | 1.0812 | -0.11 | 0.9134 |
|  | **Odds Ratio Controlled Direct Effect (CDE)** | 0.982 | 0.05667 | 0.871 | 1.0931 | -0.32 | 0.7513 |
|  | **Odds Ratio Natural Direct Effect (NDE)** | 0.996 | 0.04269 | 0.9123 | 1.0797 | -0.09 | 0.9253 |
|  | **Odds Ratio Natural Indirect Effect (NIE)** | 0.9992 | 0.01053 | 0.9786 | 1.0199 | -0.07 | 0.9416 |
| IAD of oVEMP | **Odds Ratio Total Effect** | 3.0722 | 3.1677 | -3.1363 | 9.2808 | 0.65 | 0.513 |
|  | **Odds Ratio Controlled Direct Effect (CDE)** | 1.3102 | 1.1558 | -0.9551 | 3.5755 | 0.27 | 0.7884 |
|  | **Odds Ratio Natural Direct Effect (NDE)** | 2.4488 | 2.3339 | -2.1255 | 7.0231 | 0.62 | 0.5347 |
|  | **Odds Ratio Natural Indirect Effect (NIE)** | 1.2546 | 0.4439 | 0.3846 | 2.1246 | 0.57 | 0.5663 |
| p13 latency | **Odds Ratio Total Effect** | 0.9059 | 0.1514 | 0.6092 | 1.2026 | -0.62 | 0.5343 |
|  | **Odds Ratio Controlled Direct Effect (CDE)** | 0.9883 | 0.1455 | 0.7032 | 1.2734 | -0.08 | 0.9359 |
|  | **Odds Ratio Natural Direct Effect (NDE)** | 0.9153 | 0.149 | 0.6233 | 1.2073 | -0.57 | 0.5695 |
|  | **Odds Ratio Natural Indirect Effect (NIE)** | 0.9898 | 0.03805 | 0.9152 | 1.0644 | -0.27 | 0.7885 |
| Normalized p13–n23 amplitude | **Odds Ratio Total Effect** | 0.76 | 0.1571 | 0.4521 | 1.0679 | -1.53 | 0.1266 |
|  | **Odds Ratio Controlled Direct Effect (CDE)** | 0.8362 | 0.1084 | 0.6238 | 1.0487 | -1.51 | 0.1308 |
|  | **Odds Ratio Natural Direct Effect (NDE)** | 0.765 | 0.1578 | 0.4558 | 1.0743 | -1.49 | 0.1365 |
|  | **Odds Ratio Natural Indirect Effect (NIE)** | 0.9934 | 0.025 | 0.9444 | 1.0424 | -0.26 | 0.792 |
| IAD of cVEMP | **Odds Ratio Total Effect** | 1.0121 | 0.009015 | 0.9944 | 1.0297 | 1.34 | 0.1806 |
|  | **Odds Ratio Controlled Direct Effect (CDE)** | 1.0159 | 0.01084 | 0.9947 | 1.0372 | 1.47 | 0.1418 |
|  | **Odds Ratio Natural Direct Effect (NDE)** | 1.0052 | 0.009567 | 0.9864 | 1.0239 | 0.54 | 0.5883 |
|  | **Odds Ratio Natural Indirect Effect (NIE)** | 1.0069 | 0.003936 | 0.9991 | 1.0146 | 1.74 | 0.0815 |

*Mediation analysis is a methodology used to understand how an independent variable (X) transmits its effect to an outcome (Y) through a mediator (M; H&Y scale < 3.0 vs. $\geq$3.0).

AC = anterior canal, cVEMP = cervical vestibular-evoked myogenic potential, FOG = freezing of gait, HC = horizontal canal, H&Y = Hoehn and Yahr, IAD = interaural difference, oVEMP = ocular VEMP, PC = posterior canal, VOR = vestibulo-ocular reflex
